# Supplementary material for: Aberrant enhancer hypomethylation contributes to hepatic carcinogenesis through global transcriptional reprogramming
Source: Nat Commun. 2019 Jan 18;10:335. doi: 10.1038/s41467-018-08245-z (PMC6338783; doi:10.1038/s41467-018-08245-z)
Supplement: Supplementary file 1 — Supplementary Information [file 41467_2018_8245_MOESM1_ESM.pdf]

Supplementary Information

Aberrant Enhancer Hypomethylation Contributes to Hepatic Carcinogenesis through Global Transcriptional Reprogramming

Xiong *et al.*

Supplementary Figure Legends

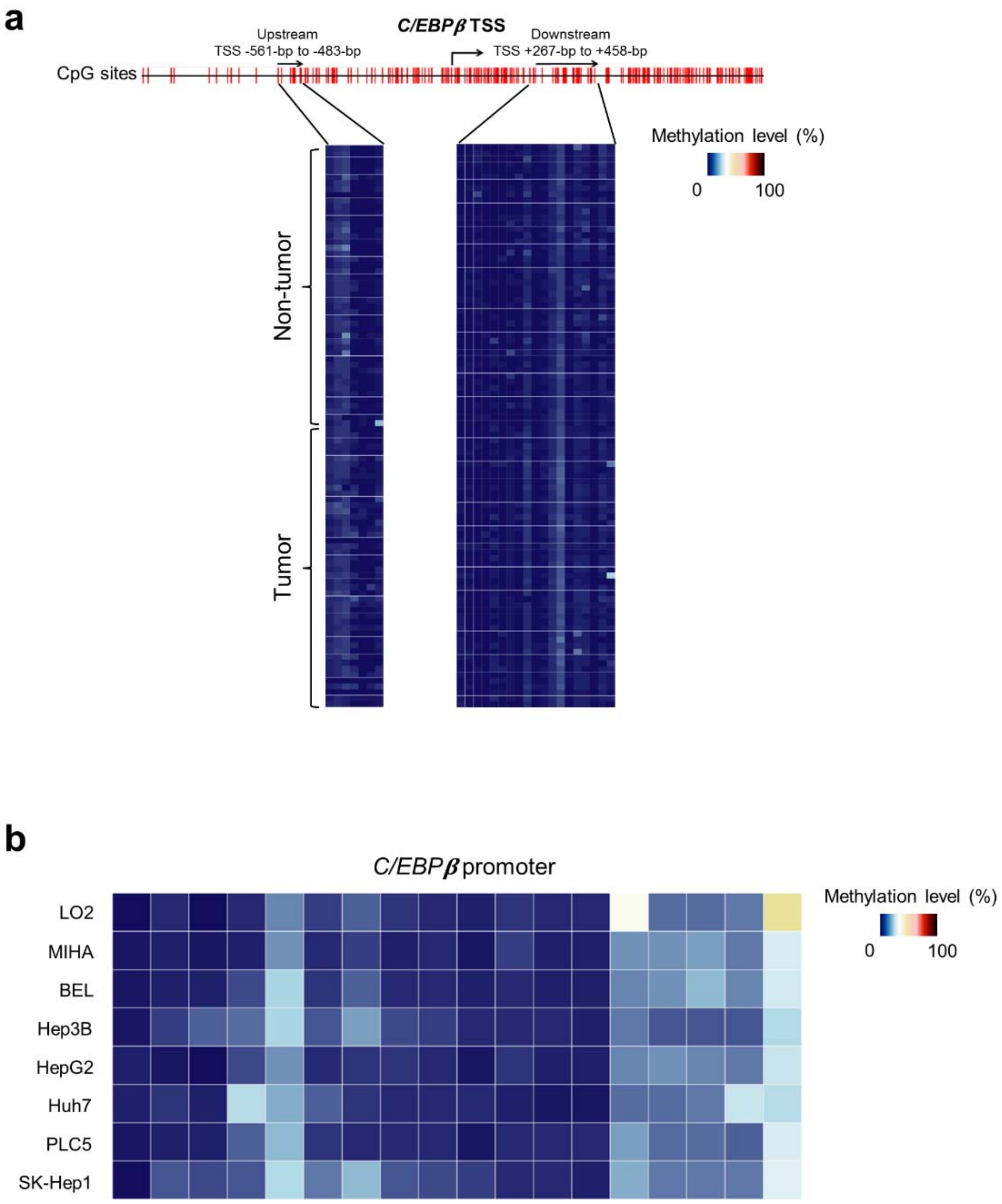

Supplementary Figure 1. Low *C/EBPβ* promoter methylation level in human HCC. (a-b) Pyrosequencing analysis of (a) 48 pairs of HCC tumor and non-tumor tissues (upstream and downstream of *C/EBPβ* TSS) and (b) 8 liver cell lines (downstream of *C/EBPβ* TSS).

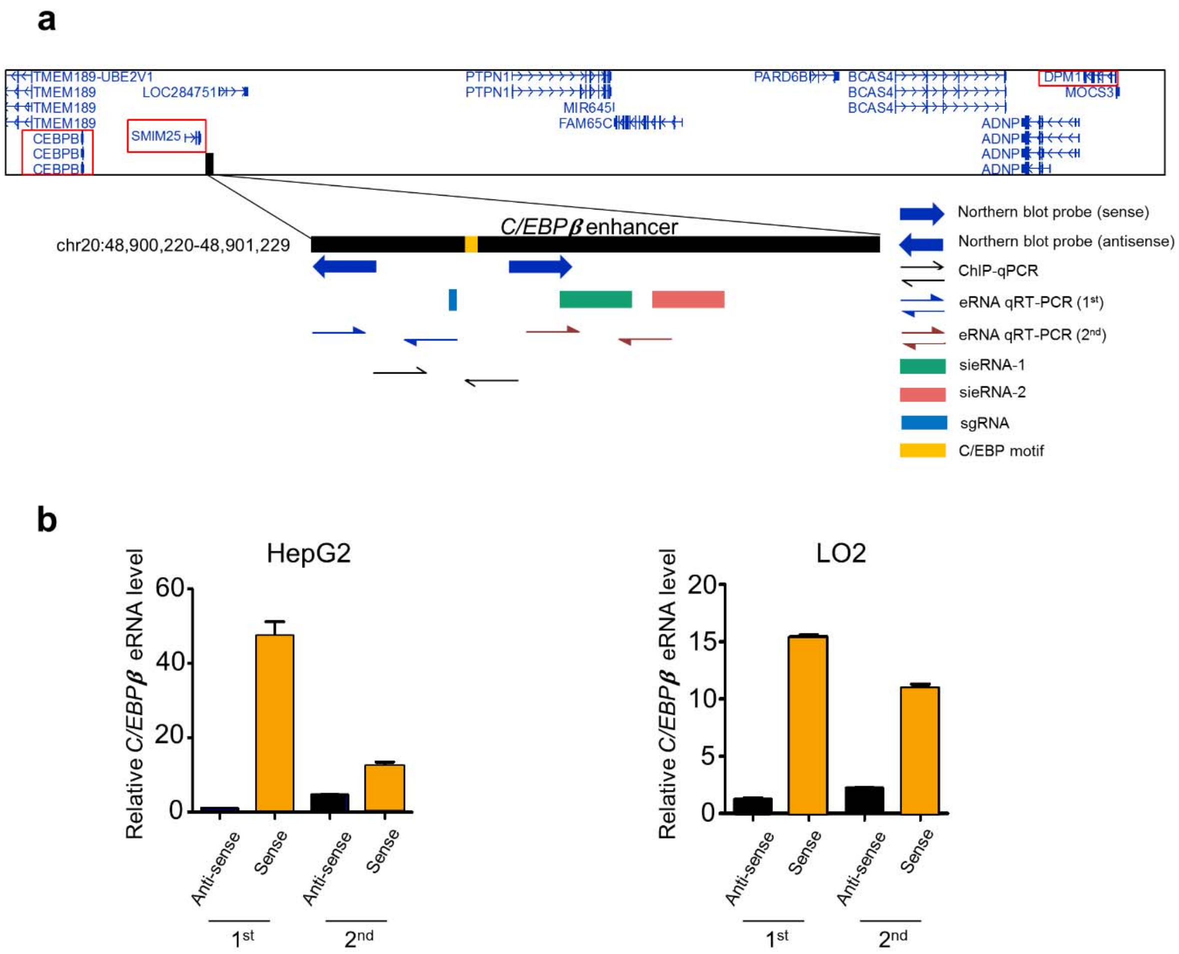

Supplementary Figure 2. Molecular analyses of *C/EBPβ* enhancer. (a) The binding positions of Northern blot probes, ChIP-qPCR primers, eRNA qRT-PCR primers, sgRNA, sieRNA-1 and sieRNA-2 at *C/EBPβ* enhancer containing the C/EBP motif are indicated in the diagram. (b) qRT-PCR analysis of *C/EBPβ* eRNA levels in the anti-sense and sense strands of two liver cell lines using two sets of primers. *C/EBPβ* eRNA levels were calculated by the $2^{-\Delta\Delta Ct}$ method using 18s rRNA as internal control, and are presented as fold-changes against the average values of the respective anti-sense groups. As the qPCR signals generated from the anti-sense strand template were much lower than those from the sense strand, these findings suggest that *C/EBPβ* eRNA transcription was unidirectional. Data are presented as mean ± SD.

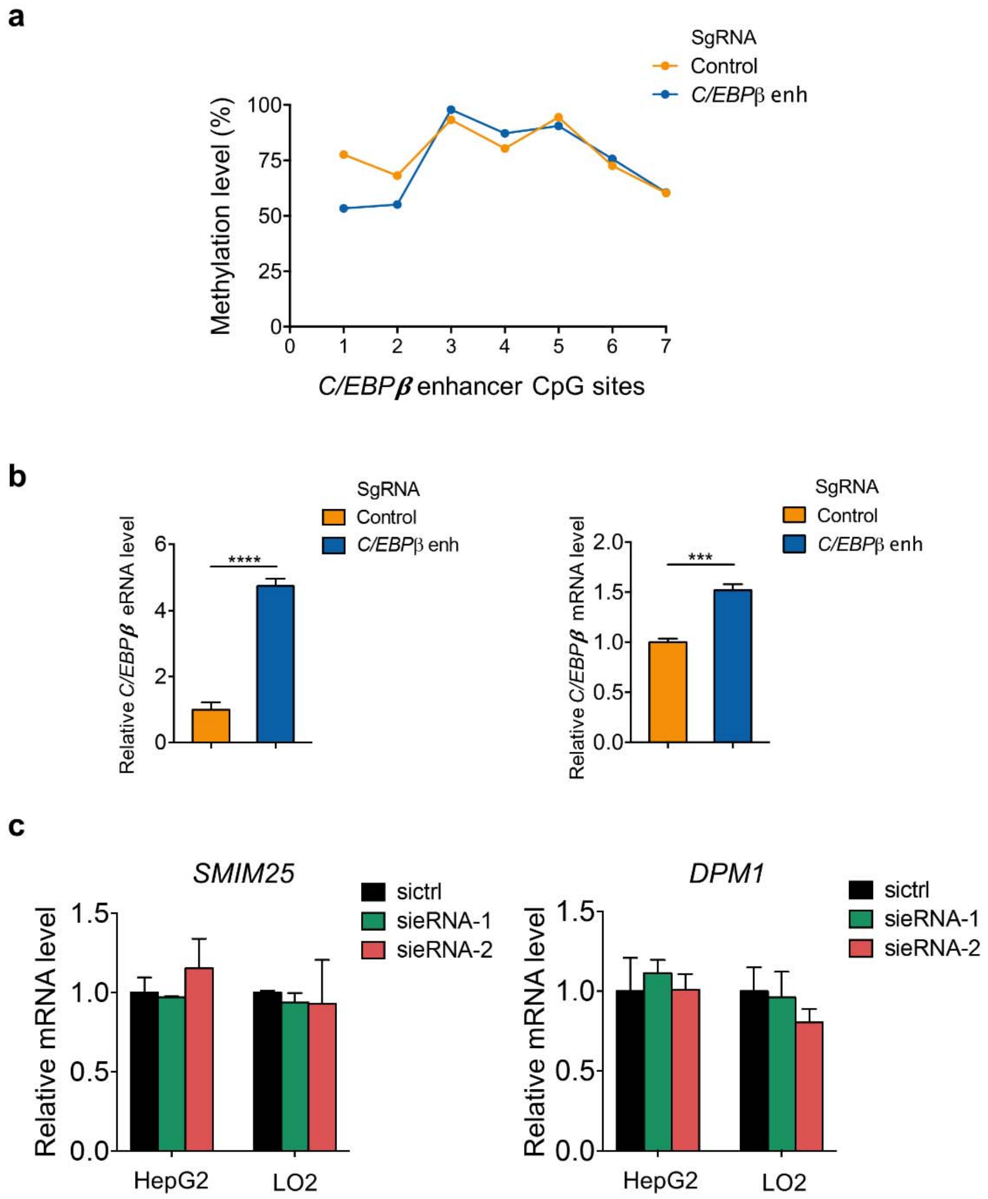

Supplementary Figure 3. (a) Pyrosequencing and (b) qRT-PCR analyses of SK-Hep1 cells transfected with pPlatTET-gRNA2 and sgRNA-expressing vectors targeting control sequence or *C/EBPβ* enhancer. Methylation levels of 7 CpG sites in *C/EBPβ* enhancer region 1 as depicted in Fig. 1g are shown. *C/EBPβ* eRNA/mRNA levels were calculated by the $2^{-\Delta\Delta Ct}$ method using 18s rRNA as internal control, and are presented as fold-changes against the average values of the respective sgRNA control groups. (c) qRT-PCR analysis of *SMIM25* and *DPM1* (located upstream and downstream of *C/EBPβ* enhancer) mRNA expressions in two

liver cell lines upon siRNA-mediated knockdown of *C/EBPβ* eRNA. The mRNA levels were calculated by the $2^{-\Delta\Delta Ct}$ method using 18s rRNA as internal control, and are presented as fold-changes against the average values of the respective siCtrl groups. Data are presented as mean ± SD. ***$P < 0.001$; ****$P < 0.0001$ as calculated by unpaired two-tailed Student's *t*-test (b).

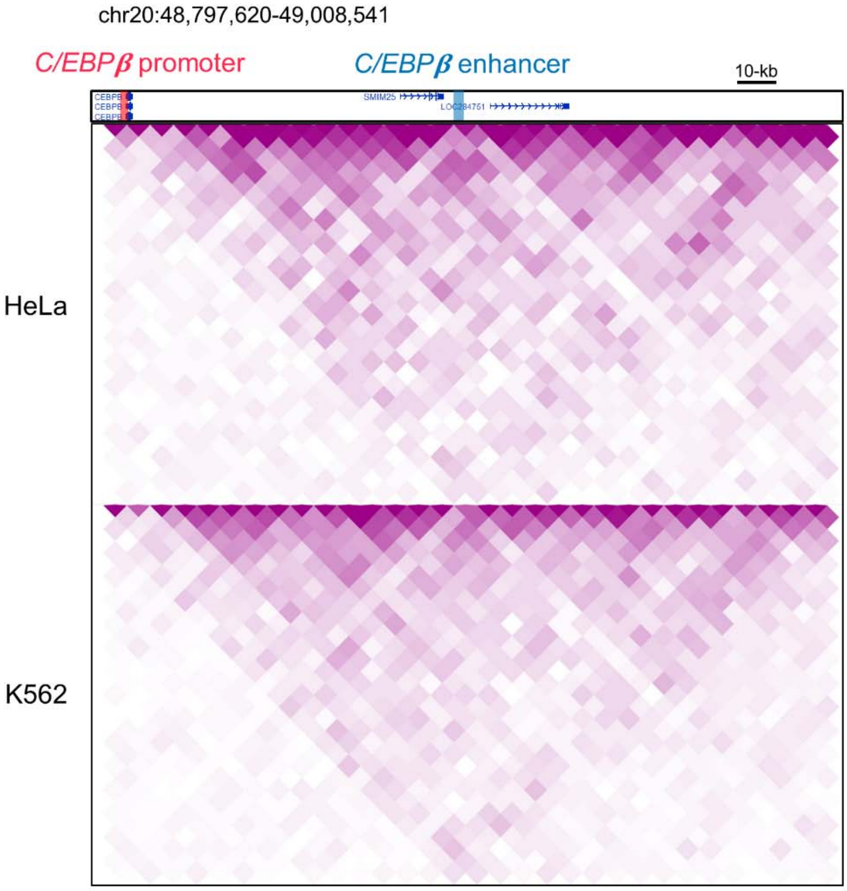

Supplementary Figure 4. *C/EBPβ* promoter and enhancer interaction based on Hi-C data in two biological replicates of human cell lines, HeLa (upper panel) and K562 (lower panel), visualized with bin size of 5-kb. Visualization of Hi-C signals predicts the span of topologically-associating domains with distinguished chromatin interactions.

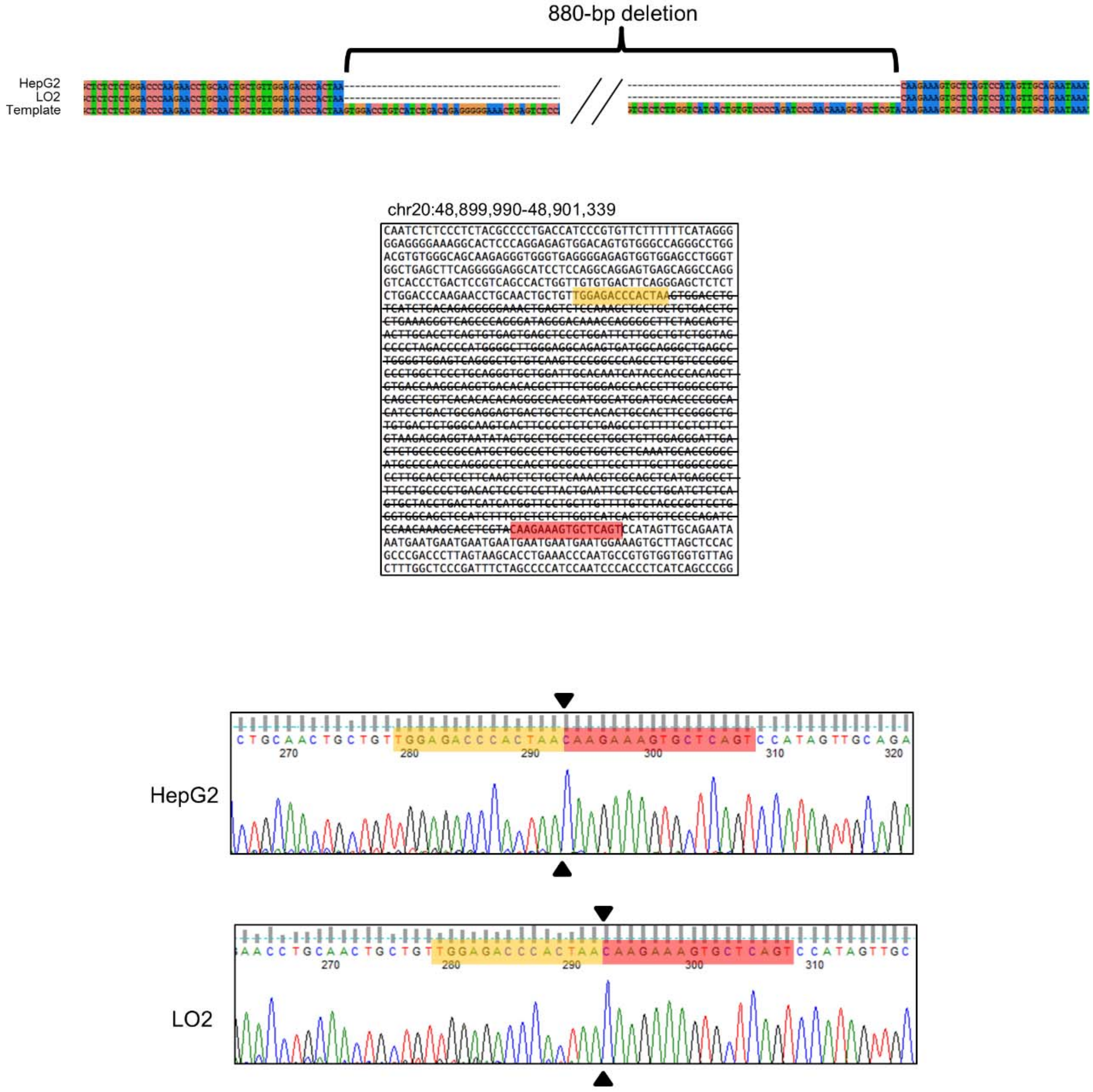

Supplementary Figure 5. Validation of CRISPR/Cas9-mediated deletion of *C/EBPβ* enhancer in two liver cell lines by Sanger sequencing.

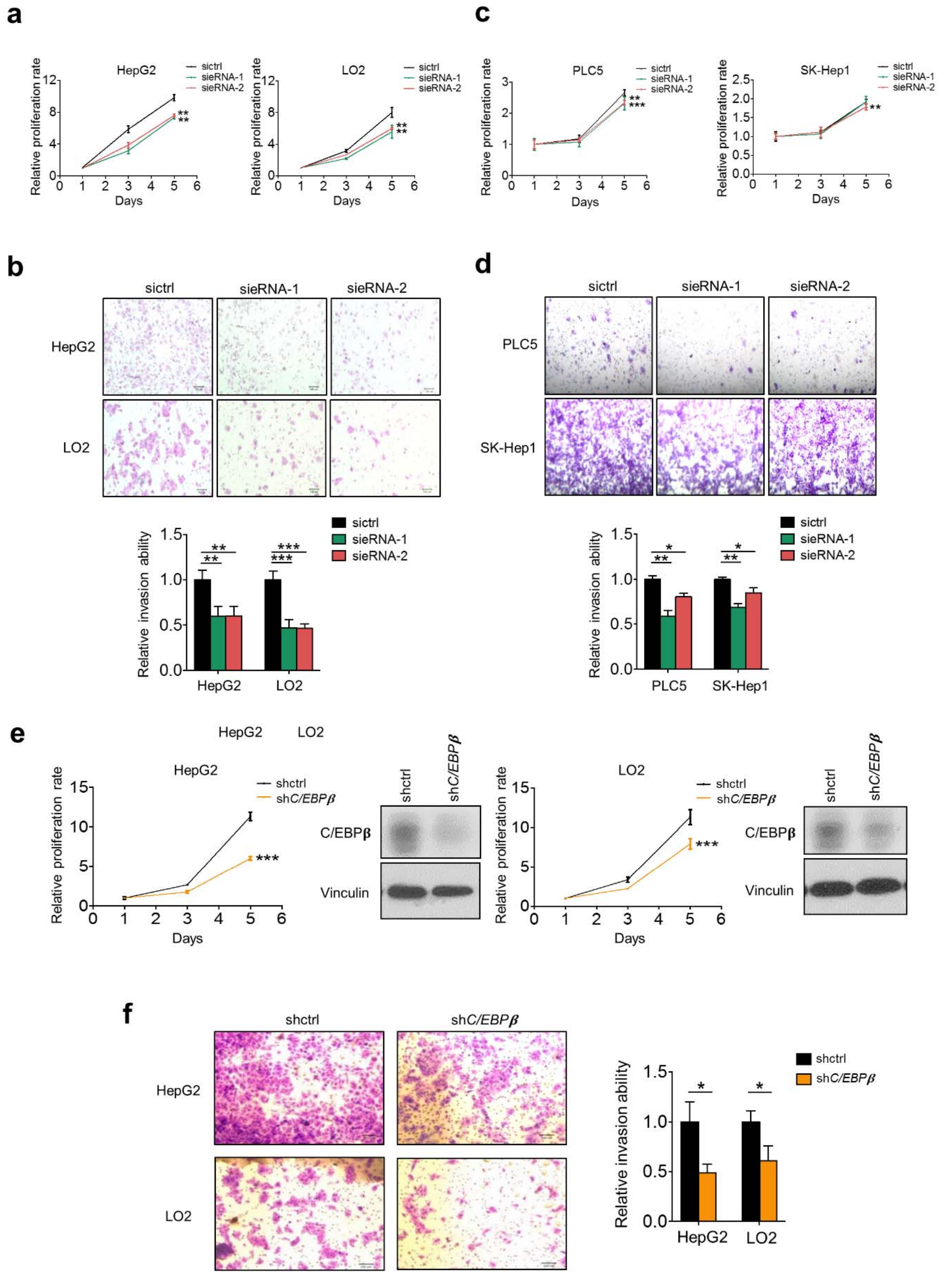

Supplementary Figure 6. Functional significance of *C/EBPβ* eRNA and mRNA in HCC. (a-b) siRNA-mediated knockdown of *C/EBPβ* eRNA in HepG2 and LO2 liver cells impaired (a)

cellular proliferation and (b) cell invasion determined by MTS assay and Matrigel chambers, respectively. (c-d) In contrast, *C/EBPβ* eRNA knockdown in PLC5 and SK-Hep1 liver cells modestly reduced (c) cellular proliferation and (d) cell invasion determined by MTS assay and Matrigel chambers, respectively. (e-f) shRNA-mediated knockdown of *C/EBPβ* mRNA in liver cells decreased (e) cellular proliferation and (f) cell invasion determined by MTS assay and Matrigel chambers, respectively. Western blot analysis of C/EBPβ level in HepG2 and LO2 cells. Vinculin was used as loading control. Representative images of Gentian violet-stained invaded cells are shown in b, d and f. Data are presented as mean ± SD. *$P < 0.05$; **$P < 0.01$; ***$P < 0.001$ as calculated by two-way ANOVA (a, c), and unpaired two-tailed Student's t-test (b, d-f).

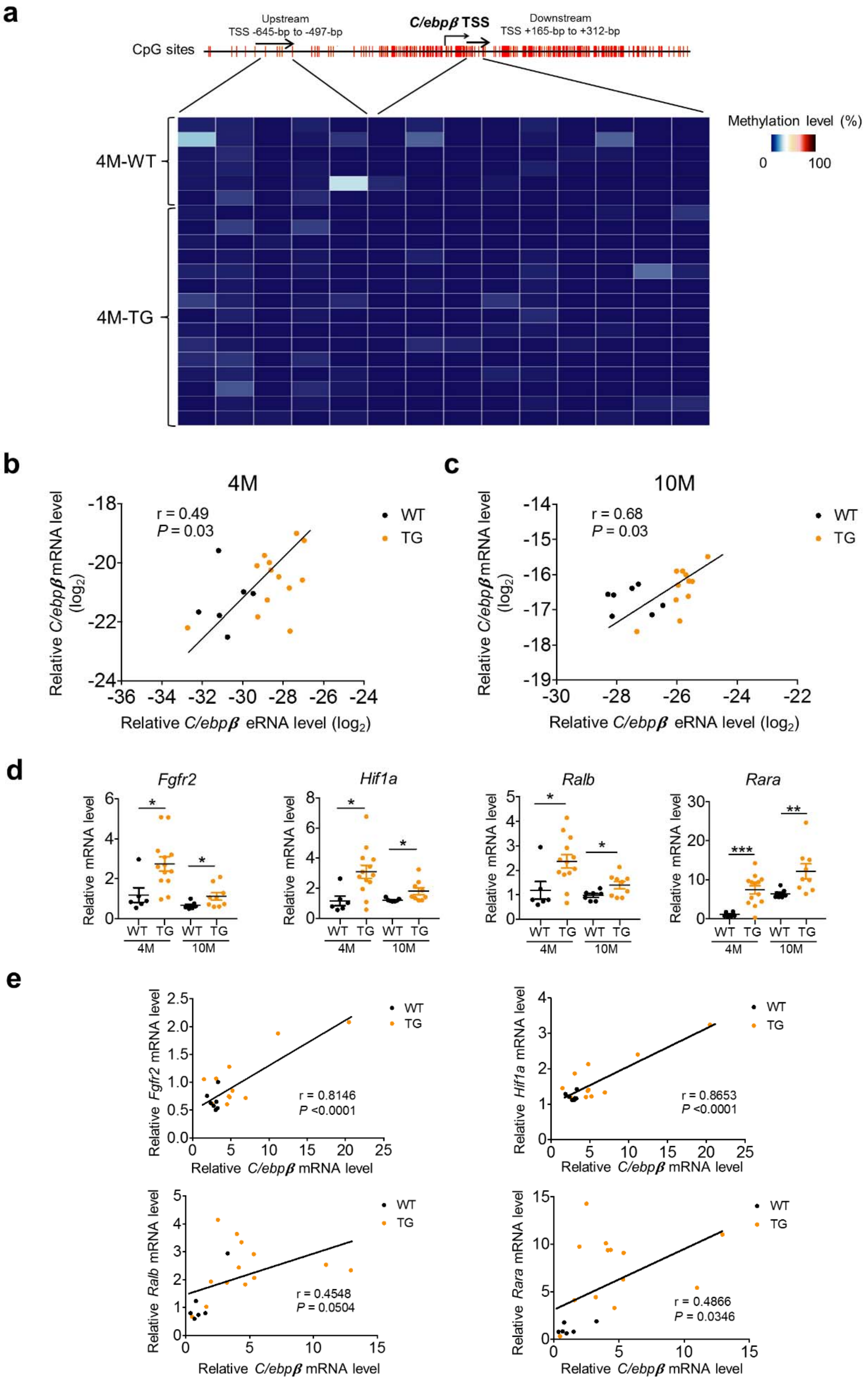

Supplementary Figure 7. Pyrosequencing and qRT-PCR analyses of murine *C/ebpβ* enhancer methylation, eRNA and mRNA expressions. (a) Methylation levels at individual CpG sites of

C/ebpβ promoter of liver tissues of 4-month-old WT and HBx TG mice by pyrosequencing. (b-c) Correlation between *C/ebpβ* eRNA and mRNA levels of (b) 4- and (c) 10-month-old WT and HBx TG mice denoted with Spearman correlation coefficients. (b-c) *C/ebpβ* mRNA levels are ΔCt values using 18s rRNA as internal control. (d) qRT-PCR analyses of *Fgfr2*, *Hif1a*, *Ralb*, and *Rara* expressions in the liver tissues of 4- and 10-month-old WT and HBx TG mice. The mRNA levels were calculated by the $2^{-\Delta\Delta Ct}$ method using 18s rRNA as internal control, and are presented as fold-changes against the average value of the 4-month-old WT group. (e) Correlations between the expressions of *C/ebpβ* and HCC driver genes in the liver tissues of WT and HBx TG mice denoted with Pearson correlation coefficient (r). The mRNA levels of *Ralb*/*Rara* and *Fgfr2*/*Hif1a* in 4- and 10-month-old mice, respectively, were calculated as in (d). *$P < 0.05$; **$P < 0.01$; ***$P < 0.001$ as calculated by Pearson correlation test (b, c, e), and unpaired two-tailed Student's t-test (d).

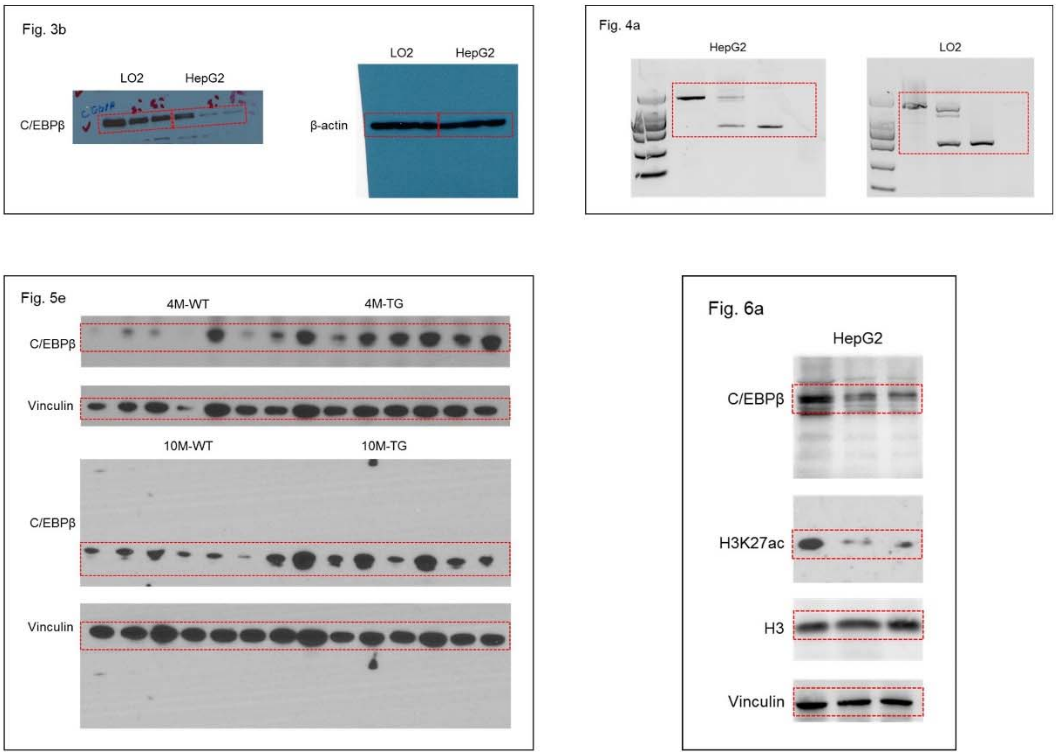

Supplementary Figure 8. Uncropped and unprocessed Western Blots. Dot line boxes indicate the cropped areas shown in the corresponding figures.

Supplementary Table 1. Integrative epigenomic analysis reveals differentially-methylated enhancers in human HCCs

Enhancer location	Methylation changes[a]	Target gene[b]	Expression changes[c]	eRNA-mRNA correlation[d]
chr8:142105625-142105840	-0.50	*SLC45A4*	3.397349521	0.816
chr8:142237099-142237665	-0.24			0.836
chr16:11707277-11708060	-0.47	*LITAF*	1.419832002	0.803
chr16:11692306-11692793	-0.24			0.83
chr16:11705983-11706695	-0.16			0.801
chr20:35964135-35964688	-0.37	*SRC*	3.886822647	0.802
chr21:34752926-34753284	-0.36	*IFNGR2*	2.514799874	0.839
chr3:182928659-182929484	-0.28	*B3GNT5*	7.516777301	0.819
chr1:213090277-213090696	-0.26	*FLVCR1*	5.552759099	0.866
chr15:81315785-81316530	-0.25	*MESDC1*	1.723013559	0.883
chr6:13302642-13303798	-0.25	*TBC1D7*	3.331748742	0.83
chr16:87987504-87988357	-0.22	*BANP*	1.215070263	0.891
chr20:48900220-48901229	-0.21	*C/EBPβ*	1.271349807	0.863
chr20:48888419-48889173	-0.19			0.85
chr20:48887757-48888308	-0.19			0.803
chr2:10471190-10471549	-0.19	*HPCAL1*	2.091328331	0.853
chr20:47390467-47391044	-0.19	*PREX1*	1.739291664	0.829
chr20:47376585-47377468	-0.19			0.811
chr21:45566076-45566451	-0.18	*ICOSLG*	1.651557117	0.817
chr3:10238203-10238635	-0.17	*IRAK2*	4.328592295	0.839
chr1:27160002-27160387	-0.15	*ZDHHC18*	2.195928042	0.948
chr6:44230828-44231731	-0.12	*NFKBIE*	2.145296308	0.868
chr8:56798106-56798776	-0.11	*LYN*	2.154508688	0.896
chr3:11314967-11316155	-0.11	*ATG7*	1.299000289	0.815
chr17:38482426-38483282	-0.10	*RARA*	1.711086537	0.857
chr21:40182920-40183779	0.11	*ETS2*	0.331982605	0.852
chr13:72438548-72439003	0.11	*DACH1*	0.067484498	0.823

Footnotes:

a. Average difference of beta value between tumor and normal liver samples

b. Target gene based on FANTOM5 database

c. Tumor vs. matched non-tumor ratio

d. Expression correlation based on FANTOM5 database

Supplementary Table 2. Clinicopathological information of the HCC patients

HCC patient samples used for genome-wide methylation analysis									
**Patient no.**	**Sex**	**Age**	**HBsAg**	**α-HCV**	**NAFLD**	**Differentiation**	**AJCC**	**Cirrhosis**	**Fibrosis**
190	male	51	positive	negative	negative	moderate	3	yes	no
293	male	45	positive	n/a	negative	moderate	1	no	yes
304	female	67	positive	n/a	negative	moderate	1	yes	no
318	male	32	positive	negative	negative	poor	3	no	no
321	male	66	positive	negative	negative	poor	2	no	yes
328	male	51	positive	negative	negative	moderate	3	yes	yes
333	female	67	positive	negative	negative	well	2	yes	no
339	female	65	positive	negative	negative	well	1	no	yes
350	male	72	negative	negative	negative	moderate	1	no	no
353	male	73	negative	positive	negative	moderate	3	yes	yes
391	male	66	positive	negative	negative	poor	3	yes	yes
414	female	72	positive	n/a	negative	poor	1	yes	n/a
419	female	78	negative	negative	negative	moderate	1	no	no
432	male	33	positive	n/a	negative	poor	3	no	yes
433	male	40	positive	negative	negative	moderate	3	yes	yes
434	female	45	positive	n/a	negative	well	1	no	yes
442	male	72	positive	negative	negative	moderate	2	yes	yes
447	female	71	positive	n/a	negative	moderate	2	yes	yes
458	male	68	negative	positive	negative	well	1	yes	yes
464	male	68	positive	n/a	negative	well	1	yes	no
469	female	67	negative	negative	negative	moderate	1	yes	yes
485	male	43	positive	positive	negative	moderate	2	yes	yes
495	female	60	positive	n/a	negative	well	1	no	yes
506	male	57	positive	negative	negative	moderate	3	yes	yes
524	male	39	positive	n/a	negative	n/a	2	yes	no
531	male	68	positive	negative	negative	well	1	no	no
534	female	45	positive	negative	negative	moderate	2	no	no
551	male	70	positive	n/a	negative	moderate	1	no	no
663	male	63	positive	negative	negative	moderate	1	yes	no
672	male	63	positive	negative	negative	moderate	3	yes	no
675	male	63	positive	negative	negative	well	1	yes	no
676	male	61	positive	negative	negative	moderate	3	no	no
688	female	63	positive	negative	negative	moderate	1	yes	no
705	male	75	negative	negative	positive	moderate	3	yes	n/a
741	female	74	negative	negative	positive	moderate	3	no	n/a
768	male	55	negative	negative	positive	moderate	3	yes	n/a

Supplementary Table 2. (continued)

HCC patient samples used for pyrosequencing									
**Patient no.**	**Sex**	**Age**	**HBsAg**	**α-HCV**	**NAFLD**	**Differentiation**	**AJCC**	**Cirrhosis**	**Fibrosis**
213	male	36	positive	n/a	negative	moderate	1	no	yes
214	male	58	positive	n/a	negative	poor	1	no	yes
285	male	50	positive	negative	negative	moderate	2	no	yes
305	male	68	positive	negative	negative	poor	1	no	yes
306	male	40	positive	n/a	negative	moderate	1	no	yes
313	male	43	positive	n/a	negative	poor	1	yes	no
315	male	60	positive	negative	negative	poor	1	no	yes
323	male	65	positive	n/a	negative	moderate	1	yes	no
324	male	64	positive	n/a	negative	moderate	1	no	yes
329	male	56	positive	negative	negative	moderate	1	yes	yes
332	male	45	positive	n/a	negative	moderate	1	no	yes
338	male	59	positive	negative	negative	moderate	1	yes	yes
376	male	36	positive	negative	negative	well	1	no	yes
391	male	66	positive	negative	negative	poor	3	yes	yes
395	male	55	positive	negative	negative	moderate	2	no	yes
396	male	70	positive	n/a	negative	moderate	1	yes	n/a
412	male	27	positive	n/a	negative	moderate	1	no	yes
418	male	59	positive	negative	negative	moderate	1	no	yes
425	male	38	positive	n/a	negative	moderate	1	no	yes
427	male	53	positive	negative	negative	moderate	1	no	yes
435	male	60	positive	negative	negative	moderate	1	yes	yes
437	male	50	positive	n/a	negative	moderate	1	no	yes
441	male	59	positive	n/a	negative	moderate	1	no	yes
443	male	65	positive	n/a	negative	moderate	2	yes	no
444	male	42	positive	n/a	negative	moderate	1	yes	yes
463	male	43	positive	negative	negative	poor	1	no	yes
488	male	60	positive	negative	negative	moderate	3	yes	no
493	male	50	positive	n/a	negative	moderate	1	no	n/a
498	male	47	positive	n/a	negative	moderate	1	yes	yes
499	male	53	positive	negative	negative	moderate	1	no	no
500	male	49	positive	n/a	negative	moderate	1	yes	yes
512	male	48	positive	n/a	negative	moderate	1	yes	no
515	male	60	positive	n/a	negative	moderate	3	yes	yes
524	male	39	positive	n/a	negative	n/a	2	yes	no
529	male	59	positive	negative	negative	moderate	1	no	no
531	male	68	positive	negative	negative	well	1	no	no
548	male	56	positive	n/a	negative	moderate	2	yes	no
564	male	61	positive	n/a	negative	moderate	1	yes	no
566	male	51	positive	negative	negative	moderate	1	yes	yes
567	male	42	positive	n/a	negative	moderate	2	yes	no
570	male	43	positive	negative	negative	moderate	1	no	yes
581	male	50	positive	n/a	negative	moderate	3	yes	no
588	male	77	positive	n/a	negative	well	1	yes	no
591	male	62	positive	negative	negative	moderate	1	no	yes
593	male	50	N/A	n/a	negative	moderate	1	yes	yes
597	male	49	positive	n/a	negative	moderate	3	no	no
620	male	58	positive	n/a	negative	moderate	1	yes	n/a
655	male	66	positive	negative	negative	moderate	1	yes	no

Supplementary Table 3. Primer sequences

Primer name	Species	Sequence 5'-3'	Application
CEBPB-F	human	AGAAGACCGTGGACAAGCACAG	qRT-PCR
CEBPB-R	human	CTCCAGGACCTTGTGCTGCGT	qRT-PCR
CEBPB-eRNA-F	human	TGACTCTGGGCAAGTCACTT	qRT-PCR
CEBPB-eRNA-R	human	GGCAGAGTCAATCCCTCCAA	qRT-PCR
18s-rRNA-F	human	CAGCCACCCGAGATTGAGCA	qRT-PCR
18s-rRNA-R	human	TAGTAGCGACGGGCGGTGTG	qRT-PCR
SMIM25-F	human	GGTGGGGATTTTTGTGTGTT	qRT-PCR
SMIM25-R	human	GGAGAGGGGATTTCTGGAAG	qRT-PCR
DPM1-F	human	GTCTCTGGAACTCGCTACAAAGG	qRT-PCR
DPM1-R	human	ATCAGATGCTCCTGGTCTCAGC	qRT-PCR
Cebpb-F	mouse	CAACCTGGAGACGCAGCACAAG	qRT-PCR
Cebpb-R	mouse	GCTTGAACAAGTTCCGCAGGGT	qRT-PCR
Fgfr2-F	mouse	GTCTCCGAGTATGAGTTGCCAG	qRT-PCR
Fgfr2-R	mouse	CCACTGCTTCAGCCATGACTAC	qRT-PCR
Hif1a-F	mouse	CCTGCACTGAATCAAGAGGTTGC	qRT-PCR
Hif1a-R	mouse	CCATCAGAAGGACTTGCTGGCT	qRT-PCR
Ralb-F	mouse	GGTGTGCAGTACGTGGAGACAT	qRT-PCR
Ralb-R	mouse	GCTTTTCCTGCCGTTCTTGTCC	qRT-PCR
Rara-F	mouse	GCTTCCAGTCAGTGGTTACAGC	qRT-PCR
Rara-R	mouse	CAAAGCAAGGCTTGTAGATGCGG	qRT-PCR
18s-rRNA-F	mouse	GTAACCCGTTGAACCCCATT	qRT-PCR
18s-rRNA-R	mouse	CCATCCAATCGGTAGTAGCG	qRT-PCR

Supplementary Table 4. Sequencing qualities of ChIP samples

Sample	Antibody	Total reads	Mapped reads	Peaks	NSC	RSC
HepG2 *C/EBPβ* enh$^{-/-}$	H3K27ac	27,314,264	27,314,264	54,328	1.554263	1.082269
HepG2 WT	H3K27ac	25,579,504	25,579,504	57,481	1.292644	1.06017
HepG2 *C/EBPβ* enh$^{-/-}$	CEBPB	24,966,010	24,966,010	16,345	1.02207	1.172833
HepG2 WT	CEBPB	26,030,358	26,030,358	19,240	1.031545	1.219591
HepG2 *C/EBPβ* enh$^{-/-}$	BRD4	26,379,848	26,379,848	24,954	1.019824	1.108922
HepG2 WT	BRD4	26,030,358	26,030,358	19,240	1.031545	1.219591

Supplementary Methods

MBDCap-seq

Methylated DNA was eluted by the MethylMiner Methylated DNA Enrichment Kit (Invitrogen) according to the manufacturer's instructions. MBDCap libraries for sequencing were prepared following standard protocols as described previously[1]. Sequencing was done using the Illumina Genome Analyzer II (GA II) up to 36 cycles for mapping to the human genome reference sequence. Image analysis and base calling were carried out with the standard Illumina pipeline. Raw tags were aligned to human reference genome hg19 by Bowtie2 v2.0.0-beta6[2]. DMRs were identified by the R package MEDIPS v1.30.0[3] after removing known copy number variation regions in HCC from our and other published studies[4, 5, 6, 7]. Functional annotation of the DMRs was performed by the R package Annotatr v1.4.0[8].

WGBS

Raw reads were aligned to human reference genome hg19 by Bismark v0.14.3[9]. DMRs were detected by Metilene v0.2-6[10]. We further filtered the DMRs by 1) length (>300-bp long), 2) CpG number (at least 8 CpG sites), 3) coverage (>10x), and 4) an average difference of beta value between tumor and normal liver samples not less than 0.1. Functional annotation of the DMRs was performed by the R package Annotatr v1.4.0[8]. Circular visualization was performed by Circos v0.69-6[11]. The set of all human enhancers identified by FANTOM5[12] was used to identify DMEs, defined as enhancers with at least 10 CpG sites having 10x read coverage, and an average difference of beta value between tumor and non-tumor samples not less than 0.1.

Nanoscale chromatin profiling and data analysis

Nanoscale chromatin profiling was performed as described previously[13]. Tissues were fixed in 1% formaldehyde for 10 min at room temperature. Fixation was stopped by addition of glycine

to a final concentration of 125 nM. Tissue pieces were washed 3 times with TBSE buffer. Pulverized tissues were lysed in 100 µl of lysis buffer and sonicated for 16 cycles (30s on, 30s off) using a Bioruptor (Diagenode). The total volume of chromatin immunoprecipitation (ChIP) was 1 ml and the amount of antibody used was 2 µg. The input DNA was precleared with protein G Dynabeads (Life Technologies) for 1 h at 4°C and then incubated with antibodies conjugated protein G beads overnight at 4°C. The beads were washed 3 times with cold wash buffer. After recovery of ChIP and input DNA, whole-genome amplification was performed using the WGA4 kit (Sigma-Aldrich) and BpmI-WGA primers. Amplified DNA was digested with BpmI (New England Biolab). After that, 30 ng of the amplified DNA was used with the NEBNext ChIP-seq library prep reagent set (New England Biolab). Each library was sequenced to an average depth of 20 to 30 million raw reads on HiSeq4000 using 100-bp pair-end reads. Sequencing tags were mapped against the human reference genome using bowtie2 v2.2.9[2]. Reads were trimmed 10-bp from the front and the back to produce 80-bp. Only reads with mapQ >10 and with duplicates removed by rmdup were used for subsequent analysis. Significant peaks were called using MACS2 v2.1.0[14] and then the bedGraph files were fixed and converted to bigwig files with UCSC tools (bedClip, bed-Graph-ToBigWig; http://hgdownload.cse.ucsc.edu/downloads.html). Enhancer regions were identified by H3K27ac peaks and assigned to the nearest genes by GREAT[15]. Super-enhancers were defined on the basis of H3K27ac signal intensity and density[16, 17, 18]. Signal visualization were performed by IGV[19] and ngs.plot[20]. The primary antibodies for ChIP are CEBPB (sc-150, Santa Cruz Biotechnology, 1:100), BRD4 (39909, Active Motif, 1:500), H3K27ac (39133, Active Motif, 1:500), and Normal Rabbit IgG (2729, Cell Signaling Technology, 1:500). The sequencing qualities of ChIP samples are shown in Supplementary Table 4.

RNA-seq and data analysis

Briefly, sequencing libraries were sequenced on HiSeq4000 sequencer (Illumina). Paired-end reads (101-bp) were aligned to hg19 reference genome using aligner tophat v2.0.13[21]. Differential transcript expression pattern discovery was performed using Cufflinks in edgeR[22, 23].

Supplementary References

1. Zuo T*, et al.* Epigenetic silencing mediated through activated PI3K/AKT signaling in breast cancer. *Cancer research* **71**, 1752-1762 (2011).
2. Langmead B, Salzberg SL. Fast gapped-read alignment with Bowtie 2. *Nature methods* **9**, 357-359 (2012).
3. Lienhard M, Grimm C, Morkel M, Herwig R, Chavez L. MEDIPS: genome-wide differential coverage analysis of sequencing data derived from DNA enrichment experiments. *Bioinformatics* **30**, 284-286 (2014).
4. Wong N*, et al.* Genomic aberrations in human hepatocellular carcinomas of differing etiologies. *Clinical cancer research : an official journal of the American Association for Cancer Research* **6**, 4000-4009 (2000).
5. Jia D*, et al.* Genome-wide copy number analyses identified novel cancer genes in hepatocellular carcinoma. *Hepatology* **54**, 1227-1236 (2011).
6. Guichard C*, et al.* Integrated analysis of somatic mutations and focal copy-number changes identifies key genes and pathways in hepatocellular carcinoma. *Nature genetics* **44**, 694-698 (2012).
7. Wang K*, et al.* Genomic landscape of copy number aberrations enables the identification of oncogenic drivers in hepatocellular carcinoma. *Hepatology* **58**, 706-717 (2013).
8. Cavalcante RG, Sartor MA. annotatr: genomic regions in context. *Bioinformatics* **33**, 2381-2383 (2017).
9. Krueger F, Andrews SR. Bismark: a flexible aligner and methylation caller for Bisulfite-Seq applications. *Bioinformatics* **27**, 1571-1572 (2011).

10. Juhling F, Kretzmer H, Bernhart SH, Otto C, Stadler PF, Hoffmann S. metilene: fast and sensitive calling of differentially methylated regions from bisulfite sequencing data. *Genome research* **26**, 256-262 (2016).

11. Krzywinski M*, et al.* Circos: an information aesthetic for comparative genomics. *Genome research* **19**, 1639-1645 (2009).

12. Andersson R*, et al.* An atlas of active enhancers across human cell types and tissues. *Nature* **507**, 455-461 (2014).

13. Yao X*, et al.* VHL Deficiency Drives Enhancer Activation of Oncogenes in Clear Cell Renal Cell Carcinoma. *Cancer discovery* **7**, 1284-1305 (2017).

14. Zhang Y*, et al.* Model-based analysis of ChIP-Seq (MACS). *Genome biology* **9**, R137 (2008).

15. McLean CY*, et al.* GREAT improves functional interpretation of cis-regulatory regions. *Nature biotechnology* **28**, 495-501 (2010).

16. Hnisz D*, et al.* Super-enhancers in the control of cell identity and disease. *Cell* **155**, 934-947 (2013).

17. Loven J*, et al.* Selective inhibition of tumor oncogenes by disruption of super-enhancers. *Cell* **153**, 320-334 (2013).

18. Chapuy B*, et al.* Discovery and characterization of super-enhancer-associated dependencies in diffuse large B cell lymphoma. *Cancer cell* **24**, 777-790 (2013).

19. Robinson JT*, et al.* Integrative genomics viewer. *Nature biotechnology* **29**, 24-26 (2011).

20. Shen L, Shao N, Liu X, Nestler E. ngs.plot: Quick mining and visualization of next-generation sequencing data by integrating genomic databases. *BMC genomics* **15**, 284 (2014).

21. Kim D, Pertea G, Trapnell C, Pimentel H, Kelley R, Salzberg SL. TopHat2: accurate alignment of transcriptomes in the presence of insertions, deletions and gene fusions. *Genome biology* **14**, R36 (2013).

22. Trapnell C*, et al.* Differential gene and transcript expression analysis of RNA-seq experiments with TopHat and Cufflinks. *Nature protocols* **7**, 562-578 (2012).

23. Robinson MD, McCarthy DJ, Smyth GK. edgeR: a Bioconductor package for differential expression analysis of digital gene expression data. *Bioinformatics* **26**, 139-140 (2010).
