# Supplementary material for: Aberrant enhancer hypomethylation contributes to hepatic carcinogenesis through global transcriptional reprogramming
Source: Nat Commun. 2019 Jan 18;10:335. doi: 10.1038/s41467-018-08245-z (PMC6338783; doi:10.1038/s41467-018-08245-z)
Supplement: Supplementary file 3 — Reporting Summary [file 41467_2018_8245_MOESM3_ESM.pdf]

nature research

Corresponding author(s): Kevin Y. Yip, Alfred S. Cheng, Ka-Fai To

Reporting Summary

Nature Research wishes to improve the reproducibility of the work that we publish. This form provides structure for consistency and transparency in reporting. For further information on Nature Research policies, see Authors & Referees and the Editorial Policy Checklist.

Statistical parameters

When statistical analyses are reported, confirm that the following items are present in the relevant location (e.g. figure legend, table legend, main text, or Methods section).

n/a	Confirmed	
☐	☒	The exact sample size (n) for each experimental group/condition, given as a discrete number and unit of measurement
☐	☒	An indication of whether measurements were taken from distinct samples or whether the same sample was measured repeatedly
☐	☒	The statistical test(s) used AND whether they are one- or two-sided<br>*Only common tests should be described solely by name; describe more complex techniques in the Methods section.*
☒	☐	A description of all covariates tested
☒	☐	A description of any assumptions or corrections, such as tests of normality and adjustment for multiple comparisons
☐	☒	A full description of the statistics including central tendency (e.g. means) or other basic estimates (e.g. regression coefficient) AND variation (e.g. standard deviation) or associated estimates of uncertainty (e.g. confidence intervals)
☐	☒	For null hypothesis testing, the test statistic (e.g. F, t, r) with confidence intervals, effect sizes, degrees of freedom and P value noted<br>*Give P values as exact values whenever suitable.*
☒	☐	For Bayesian analysis, information on the choice of priors and Markov chain Monte Carlo settings
☒	☐	For hierarchical and complex designs, identification of the appropriate level for tests and full reporting of outcomes
☐	☒	Estimates of effect sizes (e.g. Cohen's d, Pearson's r), indicating how they were calculated
☐	☒	Clearly defined error bars<br>*State explicitly what error bars represent (e.g. SD, SE, CI)*

Our web collection on statistics for biologists may be useful.

Software and code

Policy information about availability of computer code

Data collection	ChIP-seq data were collected by HiSeq2500 sequencer (Illumina) using 101-bp single end reads on an average depth of 20-30 million raw reads. RNA-seq libraries were sequenced on HiSeq4000 sequencer (Illumina) using 101-bp paired-end reads.
Data analysis	We used the following software: R3.3.2and R package: edgeR_3.24.0 and ngs.plot. Other software: bowtie_2.2.9, MACS2_2.1.0, bedClip, bed-Graph-ToBigWig, tophat_2.0.13, IGV, Cufflinks and Homer_4.80.

For manuscripts utilizing custom algorithms or software that are central to the research but not yet described in published literature, software must be made available to editors/reviewers upon request. We strongly encourage code deposition in a community repository (e.g. GitHub). See the Nature Research guidelines for submitting code & software for further information.

Data

Policy information about availability of data

All manuscripts must include a data availability statement. This statement should provide the following information, where applicable:

- Accession codes, unique identifiers, or web links for publicly available datasets
- A list of figures that have associated raw data
- A description of any restrictions on data availability

All data supporting the findings of this study are available within the article and Supplementary Information, or from the corresponding author upon request. All

ChIP-seq and RNA-seq data generated in this study are deposited in the Gene Expression Omnibus (GEO) database under the accession number GSE123097, GSE123098, and GSE123099. Public sequencing datasets used in this study are: patterns of C/EBPβ eRNA (CAGE reads, FANTOM5), and C/EBPβ and H3K27ac binding in the human HepG2 genome (GEO: GSM935493) and mouse hepatocyte genome (GEO: GSM1854433). A reporting summary for this article is available as a Supplementary Information file.

Field-specific reporting

Please select the best fit for your research. If you are not sure, read the appropriate sections before making your selection.

☒ Life sciences ☐ Behavioural & social sciences ☐ Ecological, evolutionary & environmental sciences

For a reference copy of the document with all sections, see nature.com/authors/policies/ReportingSummary-flat.pdf

Life sciences study design

All studies must disclose on these points even when the disclosure is negative.

Sample size	Sample-size calculations were not required in the case of this study. Experiments were independently repeated at least twice as indicated, and mean and the standard deviation from the mean were calculated.
Data exclusions	Experiments were excluded that were technically invalid.
Replication	The replication of all experiments was successful.
Randomization	Randomization was not relevant for this study.
Blinding	Blinding was not relevant for this study.

Reporting for specific materials, systems and methods

Materials & experimental systems

n/a	Involved in the study	
☒	☐	Unique biological materials
☐	☒	Antibodies
☐	☒	Eukaryotic cell lines
☒	☐	Palaeontology
☐	☒	Animals and other organisms
☒	☐	Human research participants

Methods

n/a	Involved in the study	
☐	☒	ChIP-seq
☒	☐	Flow cytometry
☒	☐	MRI-based neuroimaging

Antibodies

Antibodies used	CEBPB (sc-150, Santa Cruz Biotechnology), BRD4 (39909, Active Motif); H3K27ac (39133, Active Motif), β-actin (8H10D10, Cell Signaling Technology), Vinculin (sc-25336, Santa Cruz Biotechnology), and H3 (4499, Cell Signaling Technology).
Validation	References: CEBPB (PMID 25616107), BRD4 (PMID 26220994), H3K27ac (PMID 30296942), β-actin (PMID 30279493), Vinculin (PMID 29581585), and H3 (PMID 30356100).

Eukaryotic cell lines

Policy information about cell lines

Cell line source(s)	LO2 (Cellosaurus, CVCL_6926), MIHA (Cellosaurus, CVCL_SA12), BEL7404 (Cellosaurus, CVCL_6568), Hep3B (ATCC, HB-8064), HepG2 (ATCC, HB-8065), Huh7 (JCRB, 0403), PLC5 (ATCC, CRL-8024) and SK-Hep1 (ATCC, HTB-52).
Authentication	Cell lines were obtained from original sources and were not further authenticated.
Mycoplasma contamination	Cell lines were free of mycoplasma.
Commonly misidentified lines (See ICLAC register)	

BEL7404, which was developed many years ago that no early clone is available for comparative authentication. This cell line was used once in this study (Fig. 2b) for comparisons of 8 cell lines.

Animals and other organisms

Policy information about studies involving animals; ARRIVE guidelines recommended for reporting animal research

Laboratory animals	Nude mice (5-6 weeks), and HBx TG mice fixed to C57BL/6 strain (4 or 10 months).
Wild animals	Nil
Field-collected samples	Nil

ChIP-seq

Data deposition

☒ Confirm that both raw and final processed data have been deposited in a public database such as GEO.

☒ Confirm that you have deposited or provided access to graph files (e.g. BED files) for the called peaks.

Data access links<br>*May remain private before publication.*	https://www.ncbi.nlm.nih.gov/geo/query/acc.cgi?acc=GSE123099
Files in database submission	fastq, bigWig
Genome browser session<br>(e.g. UCSC)	*Provide a link to an anonymized genome browser session for "Initial submission" and "Revised version" documents only, to enable peer review. Write "no longer applicable" for "Final submission" documents.*

Methodology

Replicates	2 replicates in each treatment setting for ChIP-seq and RNA-seq.
Sequencing depth	Pair-end sequencing (100bp) obtained 20-30 million reads per sample for ChIP-seq. The total number of reads and uniquely mapped reads are listed in Supplementary Table 4.
Antibodies	CEBPB (sc-150, Santa Cruz Biotechnology), BRD4 (39909, Active Motif), and H3K27ac (39133, Active Motif).
Peak calling parameters	Peak calling over input was performed with MACS2 v2.1.014 using default setting p-value threshold=0.01.
Data quality	The number of peaks and quality measures are listed in Supplementary Table 4.
Software	Only reads with mapQ >10 and with duplicates removed by rmdup were used for subsequent analysis. Significant peaks were called using MACS2 v2.1.014 and then the bedGraph files were fixed and converted to bigwig files with UCSC tools (bedClip, bed-Graph-ToBigWig; http://hgdownload.cse.ucsc.edu/downloads.html).
